# High-quality permanent draft genome sequence of the *Lebeckia* - nodulating *Burkholderia dilworthii* strain WSM3556^T^

**DOI:** 10.1186/s40793-015-0048-3

**Published:** 2015-09-19

**Authors:** Sofie E. De Meyer, Rui Tian, Rekha Seshadri, Natalia Ivanova, Amrita Pati, Victor Markowitz, Tanja Woyke, Ron Yates, John Howieson, Nikos Kyrpides, Wayne Reeve

**Affiliations:** Centre for Rhizobium Studies, Murdoch University, Murdoch, Western Australia Australia; DOE Joint Genome Institute, Walnut Creek, California USA; Biological Data Management and Technology Center, Lawrence Berkeley National Laboratory, Berkeley, California USA; Department of Agriculture and Food, South Perth, Western Australia Australia; Department of Biological Sciences, Faculty of Science, King Abdulaziz University, Jeddah, Saudi Arabia

**Keywords:** Root-nodule bacteria, Nitrogen fixation, *Betaproteobacteria*, South Africa, *Lebeckia*, GEBA-RNB

## Abstract

*Burkholderia dilworthii* strain WSM3556^T^ is an aerobic, motile, Gram-negative, non-spore-forming rod that was isolated from an effective N_2_-fixing root nodule of *Lebeckia ambigua* collected near Grotto Bay Nature Reserve, in the Western Cape of South Africa, in October 2004. This plant persists in infertile and deep sandy soils with acidic pH, and is therefore an ideal candidate for a perennial based agriculture system in Western Australia. WSM3556^T^ thus represents a potential inoculant quality strain for *L. ambigua* for which we describe the general features, together with genome sequence and annotation. The 7,679,067 bp high-quality permanent draft genome is arranged in 140 scaffolds of 141 contigs, contains 7,059 protein-coding genes and 64 RNA-only encoding genes, and is part of the GEBA-RNB project proposal.

## Introduction

Over the last decade, agricultural scientists have sought to discover perennial legumes from a wide range of natural environments to develop new plants for grazing systems [1]. It is thought that these plants might be more resilient to changing rainfall patterns, such as in the target environments of Western Australia. Here, winter rainfall has declined by 20 % in the last two decades [2], although more frequent summer rainfall events have been experienced. In the fynbos biome of South Africa, several species that offer potential for domestication have been discovered [1, 3]. These legumes are frequently nodulated by *Burkholderia* bacteria in the class *Betaproteobacteria* [3, 4]. The symbiosis between these *Burkholderia* and legumes from the genera *Lebeckia* and *Rhynchosia* fix atmospheric nitrogen to enable their cultivation on infertile soils [4–7]. *Lebeckia ambigua* is proving well adapted to Western Australia [1] because in areas where it is naturally found in South Africa the soil and climatic conditions approximate those of Western Australia.

Nodules and seeds of *L. ambigua* were collected in four expeditions to the Western Cape of South Africa between 2002 and 2007. The isolation of bacteria from these nodules gave rise to a collection of 23 strains that were identified as *Burkholderia* [3]. Unlike most of the previously studied nodulating *Burkholderia* strains, this South African group appears to associate with papilionoid forage legumes, rather than *Mimosa* species. WSM3556^T^ belongs to a subgroup of strains that were isolated in 2004 from nodules collected south west of Darling, in a natural rangeland site on the southern border of the Grotto Bay Nature Reserve [3]. The soil at the site of collection was deep sand with a pH of 6. *Burkholderia dilworthii* strain WSM3556^T^ was isolated from those nodules and is effective at fixing nitrogen with *L. ambigua* and *L. sepiaria*. The nodules formed by these symbioses are crotaloid and indeterminate [3].

WSM3556^T^ thus represents a potential inoculant quality strain for *L. ambigua*, which is being developed as a grazing legume adapted to infertile soils that receive 250–400 mm annual rainfall in southern Australia and is therefore of special interest to the RNB chapter of the GEBA project. Here we present a summary classification and a set of general features for *Burkholderia dilworthii* strain WSM3556^T^ together with the description of the permanent draft genome sequence and annotation.

## Organism information

### Classification and features

**Fig. 1:**
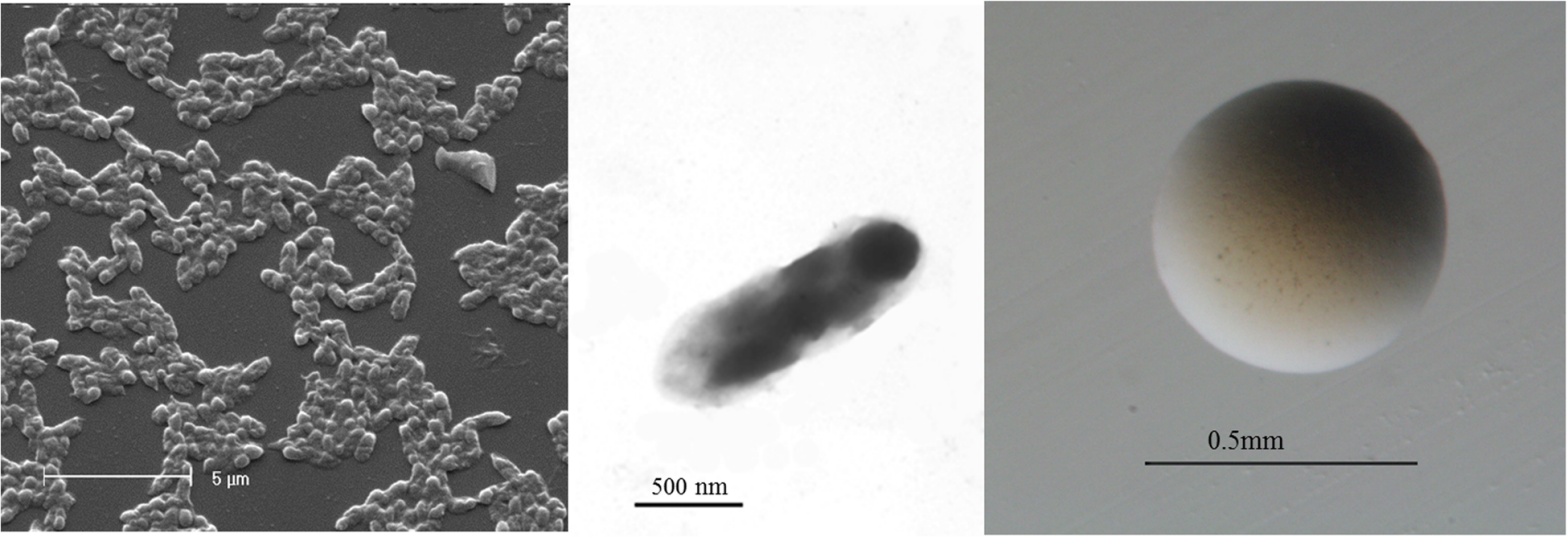
Images of *Burkholderia dilworthii* strain WSM3556^T^ using scanning (*Left*) and transmission (*Center*) electron microscopy and the appearance of colony morphology on solid media (*Right*)

*Burkholderia dilworthii* strain WSM3556^T^ is a motile, Gram-negative, non-spore-forming rod (Fig. 1 Left, Center) in the order *Burkholderiales* of the class *Betaproteobacteria*. The rod-shaped form varies in size with dimensions of 0.9–2 μm in width and 0.4–3.0 μm in length (Fig. 1 Left). It is fast growing, forming 0.4–2 mm diameter colonies after 24 h when grown on half Lupin Agar [8] and TY [9] at 28 °C. Colonies on ½LA are white-opaque, slightly domed, moderately mucoid with smooth margins (Fig. 1 Right). Additional physiological properties of this strain were previously published [5].

**Fig. 2:**
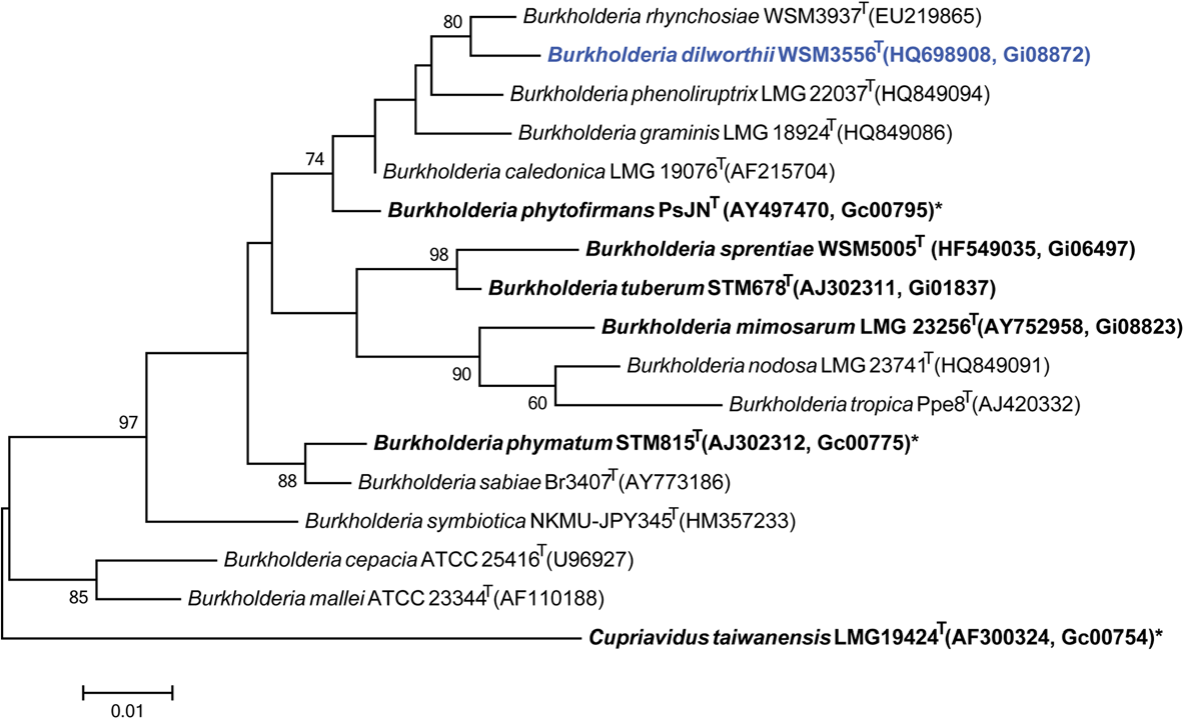
Phylogenetic tree highlighting the position of *Burkholderia dilworthii* strain WSM3556^T^ (shown in blue print), relative to other strains in the *Burkholderia* genus using a 1,322 bp internal region of the 16S rRNA gene. *Cupriavidus taiwanensis* LMG 19424^T^ was used as an outgroup. All sites were informative and there were no gap-containing sites. Phylogenetic analyses were performed using MEGA, version 5.05 [31]. The tree was build using the maximum likelihood method with the General Time Reversible model. Bootstrap analysis with 500 replicates was performed to assess the support of the clusters. Type strains are indicated with a superscript T. Strains with a genome sequencing project registered in GOLD [14] are in bold print and the GOLD ID is provided after the NCBI accession number. Published genomes are designated with an asterisk

Figure 2 shows the phylogenetic relationship of *Burkholderia dilworthii* strain WSM3556^T^ in a 16S rRNA gene sequence based tree. This strain is most similar to *Burkholderia rhynchosiae*WSM3937^T^ and *Burkholderia phytofirmans* PsJN^T^ based on the 16S rRNA with sequence identities of 98.50 % and 98.11 %, respectively, as determined using the EzTaxon-e server [10]. *Burkholderia rhynchosiae*WSM3937^T^ has been isolated from *Rhynchosia ferulifolia*, a herbaceous legume from the fynbos biome in South Africa [7]. *Burkholderia phytofirmans* PsJN^T^ was isolated from surface sterilized onion roots and has plant growth promoting properties on various plants, however it has not been reported in association with legumes [11]. Minimum Information about the Genome Sequence of WSM3556^T^ is provided in Table 1.

**Table 1:** Classification and general features of *Burkholderia dilworthii* WSM3556^T^ in accordance with the MIGS recommendations [32] published by the Genome Standards Consortium [33]

MIGS ID	Property	Term	Evidence code
	Classification	Domain *Bacteria*	TAS [34]
		Phylum *Proteobacteria*	TAS [35]
		Class *Betaproteobacteria*	TAS [36, 37]
		Order *Burkholderiales*	TAS [37, 38]
		Family *Burkholderiaceae*	TAS [37, 39]
		Genus *Burkholderia*	TAS [37, 40]
		Species *Burkholderia dilworthii*	TAS [5]
		(Type) strain: WSM3556^T^	TAS [5]
	Gram stain	Negative	TAS [5]
	Cell shape	Rod	TAS [5]
	Motility	Motile	TAS [5]
	Sporulation	Non-sporulating	TAS [39]
	Temperature range	15–37 °C	TAS [5]
	Optimum temperature	28 °C	TAS [5]
	pH range; Optimum	5.5–8; 7	TAS [5]
	Carbon source	Large range	TAS [5]
MIGS-6	Habitat	Soil, root nodule on host	IDA
MIGS-6.3	Salinity	0–10 %	TAS [5]
MIGS-22	Oxygen requirement	Aerobic	IDA
MIGS-15	Biotic relationship	Free living, symbiotic	IDA
MIGS-14	Pathogenicity	Non-pathogenic	NAS
MIGS-4	Geographic location	South Africa	TAS [3]
MIGS-5	Sample collection	2004	TAS [3]
MIGS-4.1 MIGS-4.2	Longitude	18.44	TAS [3]
	Latitude	−33.49	TAS [3]
MIGS-4.4	Altitude	237	IDA

Evidence codes – IDA: Inferred from Direct Assay; TAS: Traceable Author Statement (i.e., a direct report exists in the literature); NAS: Non-traceable Author Statement (i.e., not directly observed for the living, isolated sample, but based on a generally accepted property for the species, or anecdotal evidence). These evidence codes are from the Gene Ontology project [41]

## Symbiotaxonomy

*Burkholderia dilworthii* strain WSM3556^T^ belongs to a group of *Burkholderia* strains that nodulate papilionoid forage legumes rather than the classical *Mimosa* host species (Mimosoideae) described for other *Burkholderia* microsymbionts [12]. *Burkholderia dilworthii* strain WSM3556^T^ was assessed for nodulation and nitrogen fixation on three separate *L. ambigua* genotypes (CRSLAM-37, CRSLAM-39 and CRSLAM-41) [3]. It could nodulate and fix effectively on CRSLAM-41 but was partially effective on CRSLAM-37 and CRSLAM-39 [3]. Moreover, WSM3556^T^ also nodulates and fixes nitrogen in association with *Lebeckia sepiaria**.*

## Genome sequencing information

### Genome project history

This organism was selected for sequencing on the basis of its environmental and agricultural relevance to issues in global carbon cycling, alternative energy production, and biogeochemical importance, and is part of the Genomic Encyclopedia of Bacteria and Archaea, The Root Nodulating Bacteria chapter project at the U.S. Department of Energy, Joint Genome Institute for projects of relevance to agency missions [13]. The genome project is deposited in the Genomes OnLine Database [14] and the high-quality permanent draft genome sequence in IMG [15]. Sequencing, finishing and annotation were performed by the JGI using state of the art sequencing technology [16]. A summary of the project information is shown in Table 2.

**Table 2:** Genome sequencing project information for *Burkholderia dilworthii* WSM3556^T^

MIGS ID	Property	Term
MIGS-31	Finishing quality	High-quality-permanent-draft
MIGS-28	Libraries used	Illumina Std
MIGS-29	Sequencing platforms	Illumina HiSeq 2000
MIGS-31.2	Fold coverage	367 × Illumina
MIGS-30	Assemblers	Velvet 1.1.04, ALLPATHS V.r37348
MIGS-32	Gene calling methods	Prodigal 1.4
	Locus Tag	F759
	Genbank ID	AWZT00000000
	Genbank Date of Release	December 12, 2013
	GOLD ID	Gp0010131
	BIOPROJECT	PRJNA182743
MIGS-13	Source Material Identifier	WSM3556, LMG 27173, HAMBI3353
	Project relevance	Symbiotic N_2_fixation, agriculture

### Growth conditions and genomic DNA preparation

*Burkholderia dilworthii* strain WSM3556^T^ was grown on TY solid medium [9] for 3 days, a single colony was selected and used to inoculate 5 ml TY broth medium. The culture was grown for 48 h on a gyratory shaker (200 rpm) at 28 °C. Subsequently 1 ml was used to inoculate 60 ml TY broth medium and grown on a gyratory shaker (200 rpm) at 28 °C until OD 0.6 was reached. DNA was isolated from 60 mL of cells using a CTAB bacterial genomic DNA isolation method [17]. Final concentration of the DNA was 0.5 mg/ml.

### Genome sequencing and assembly

The genome of *Burkholderia dilworthii* strain WSM3556^T^ was sequenced at the DOE Joint Genome Institute using state of the art technology [18]. For this genome, an Illumina standard shotgun library was constructed and sequenced using the Illumina HiSeq 2000 platform, which generated 9,394,768 reads totalling 2,818.4 Mbp of Illumina data. All general aspects of library construction and sequencing performed at the JGI can be found on the JGI web site [16]. All raw Illumina sequence data was passed through DUK, a filtering program developed at JGI, which removes known Illumina sequencing and library preparation artifacts (Mingkun L, Copeland A, Han J. unpublished). The following steps were then performed for assembly: (1) filtered Illumina reads were assembled using Velvet, version 1.1.04 [19], (2) 1–3 Kbp simulated paired end reads were created from Velvet contigs using wgsim [20], (3) Illumina reads were assembled with simulated read pairs using Allpaths (version r37348) [21]. Parameters for assembly steps were: 1) Velvet -exp_cov 90 -cov_cutoff 20 -exportFiltered yes -very_clean yes), 2) wgsim (−e 0–1 76–2 76 -r 0 -R 0 -X 0 -d 3000 -s 300 -N 1266735), 3) Allpaths–LG (PrepareAllpathsInputs: PHRED_64 = 1 PLOIDY = 1 JUMP_COVERAGE = 25 FRAG_COVERAGE = 125, RunAllpathsLG: RUN = 125std + 25xfakedpairs TARGETS = standard VAPI_WARN_ONLY = True OVERWRITE = True). The final draft assembly contained 141 contigs in 140 scaffolds. The total size of the genome is 7.7 Mbp and the final assembly is based on 2,818.4 Mbp of Illumina draft data, which provides an average of 367x coverage of the genome.

### Genome annotation

Genes were identified using Prodigal [22], as part of the DOE-JGI genome annotation pipeline [23, 24] followed by a round of manual curation using GenePRIMP [25] for finished genomes and Draft genomes in fewer than 10 scaffolds. The predicted CDSs were translated and used to search the NCBI non-redundant database, UniProt, TIGRFam, Pfam, KEGG, COG, and InterPro databases. The tRNAScanSE tool [26] was used to find tRNA genes, whereas ribosomal RNA genes were found by searches against models of the ribosomal RNA genes built from SILVA [27]. Other non–coding RNAs such as the RNA components of the protein secretion complex and the RNase P were identified by searching the genome for the corresponding Rfam profiles using INFERNAL [28]. Additional gene prediction analysis and manual functional annotation was performed within the Integrated Microbial Genomes-Expert Review system [29] developed by the Joint Genome Institute, Walnut Creek, CA, USA.

## Genome properties

The genome is 7,679,067 nucleotides with 61.77 % GC content (Table 3) and comprised of 140 scaffolds and 141 contigs. From a total of 7,123 genes, 7,059 were protein encoding and 64 RNA only encoding genes. The majority of genes (76.25 %) were assigned a putative function whilst the remaining genes were annotated as hypothetical. The distribution of genes into COG functional categories is presented in Table 4.

**Table 3:** Genome statistics for *Burkholderia dilworthii* strain WSM3556^T^

Attribute	Value	% of total
Genome size (bp)	7,679,067	100.00
DNA coding (bp)	6,485,063	84.45
DNA G + C (bp)	4,743,598	61.77
DNA scaffolds	140	100.00
Total genes	7,123	100.00
Protein-coding genes	7,059	99.10
RNA genes	64	0.90
Pseudo genes	0	0.00
Genes in internal clusters	426	5.98
Genes with function prediction	5,431	76.25
Genes assigned to COGs	4,704	66.04
Genes with Pfam domains	5,730	80.44
Genes with signal peptides	642	9.01
Genes with transmembrane helices	1,585	22.25
CRISPR repeats	0	0

**Table 4:** Number of genes associated with general COG functional categories

Code	Value	% age	COG category
J	186	3.50	Translation, ribosomal structure and biogenesis
A	1	0.02	RNA processing and modification
K	528	9.94	Transcription
L	183	3.44	Replication, recombination and repair
B	1	0.02	Chromatin structure and dynamics
D	34	0.64	Cell cycle control, Cell division, chromosome partitioning
V	50	0.94	Defense mechanisms
T	235	4.42	Signal transduction mechanisms
M	310	5.83	Cell wall/membrane/envelope biogenesis
N	92	1.73	Cell motility
U	133	2.50	Intracellular trafficking, secretion, and vesicular transport
O	159	2.99	Posttranslational modification, protein turnover, chaperones
C	362	6.81	Energy production and conversion
G	445	8.38	Carbohydrate transport and metabolism
E	581	10.94	Amino acid transport and metabolism
F	89	1.68	Nucleotide transport and metabolism
H	195	3.67	Coenzyme transport and metabolism
I	255	4.80	Lipid transport and metabolism
P	262	4.93	Inorganic ion transport and metabolism
Q	179	3.37	Secondary metabolite biosynthesis, transport and catabolism
R	600	11.29	General function prediction only
S	431	8.11	Function unknown
-	2419	33.96	Not in COGS

The total is based on the total number of protein coding genes in the genome

## Conclusion

*Burkholderia dilworthii*WSM3556^T^ belongs to a group of Beta-rhizobia isolated from *Lebeckia ambigua* from the fynbos biome in South Africa [3]. WSM3556^T^ is phylogeneticaly most closely related to *Burkholderia rhynchosiae*WSM3937^T^ and *Burkholderia phytofirmans* PsJN^T^. Of these strains only WSM3556^T^ and WSM3937^T^ are legume microsymbionts. Out of 13 *Burkholderia* strains that are known legume microsymbionts, only four (WSM3556^T^, WSM4176, WSM5005^T^, STM678^T^) nodulate South African papilionoid species. A comparison of these nodulating strains reveals that WSM3556^T^ has the smallest genome (7.7 Mbp), the smallest KOG count (1295) and the lowest GC (61.77 %) percentage in this group. These four genomes share the nitrogenase-RXN MetaCyc pathway catalyzed by a multiprotein nitrogenase complex. Strains WSM3556^T^, WSM4176, WSM5005^T^ [30] have been shown to fix nitrogen with *Lebeckia ambigua* provenances with varying degrees of effectiveness. WSM3556^T^ is partially effective on two out of three *L. ambigua* provenances, WSM4176 is partially effective on only one *L. ambigua* provenance and WSM5005^T^ is effective on all three *L. ambigua* provenances. The genome sequences of these fynbos bacteria provides an unprecedented opportunity to reveal the genetic determinants required for effective nitrogen fixation with *Lebeckia*.

## Abbreviations

GEBA-RNB: Genomic Encyclopedia of Bacteria and Archaea – Root Nodule Bacteria
JGI: Joint Genome Institute
TY: Trypton yeast
CTAB: Cetyl trimethyl ammonium bromide
WSM: Western Australian Soil Microbiology
